# A hybrid approach to protein folding problem integrating constraint programming with local search

**DOI:** 10.1186/1471-2105-11-S1-S39

**Published:** 2010-01-18

**Authors:** Abu Dayem Ullah, Kathleen Steinhöfel

**Affiliations:** 1 King's College London, Department of Computer Science, London WC2R 2LS, UK

## Abstract

**Background:**

The protein folding problem remains one of the most challenging open problems in computational biology. Simplified models in terms of lattice structure and energy function have been proposed to ease the computational hardness of this optimization problem. Heuristic search algorithms and constraint programming are two common techniques to approach this problem. The present study introduces a novel hybrid approach to simulate the protein folding problem using constraint programming technique integrated within local search.

**Results:**

Using the face-centered-cubic lattice model and 20 amino acid pairwise interactions energy function for the protein folding problem, a constraint programming technique has been applied to generate the neighbourhood conformations that are to be used in generic local search procedure. Experiments have been conducted for a few small and medium sized proteins. Results have been compared with both pure constraint programming approach and local search using well-established local move set. Substantial improvements have been observed in terms of final energy values within acceptable runtime using the hybrid approach.

**Conclusion:**

Constraint programming approaches usually provide optimal results but become slow as the problem size grows. Local search approaches are usually faster but do not guarantee optimal solutions and tend to stuck in local minima. The encouraging results obtained on the small proteins show that these two approaches can be combined efficiently to obtain better quality solutions within acceptable time. It also encourages future researchers on adopting hybrid techniques to solve other hard optimization problems.

## Background

A protein folds into a state of minimum free energy called its *native state *or *tertiary structure*. As presented in [1], this folded tertiary structure can be predicted from the sequence of component amino acids known as the *primary structure*. Knowledge of final 3D structure of proteins helps to design drugs and detect structural differences due to misfolding; therefore, crucial to pharmacology and medical science. The general principle by which a natural protein folds and efficient prediction of its tertiary structure remain the most challenging problems in computational biology.

This mystery has stimulated researchers to protein folding simulations by using reliable and faster computational techniques. Various simplified models have been proposed in terms of lattice structure and energy function to ease the computational complexity of this hard problem. The prevailing strategy to solve protein folding problem has been to determine a placement of the amino acids in 3D space that results in the minimum energy structure. Energy function has been modeled that captures the idea of assigning energy between pairs of amino acids placed within a certain distance. Even with the simplest of *Hydrophobic-Polar *(HP) energy function and discrete rectangular lattice model (i.e., HP model [2]), the problem is shown NP-Complete [3]. The problem is also shown NP-hard for HP-like models on generalized lattices [4].

Effectively the protein folding problem reduces to an optimization problem where the energy function has to be minimized under a set of constraints. Consequently, one can resort to *Constraint Programming *(CP) and stochastic *Local Search *(LS) algorithms to tackle this problem. Both techniques are commonly used to approach hard optimization problems.

Local search methods generate solutions in polynomial time by doing a clever sampling in the search space of exponential size. The major drawback is that it can not guarantee optimal solutions in polynomial time since the search space is randomly explored and it often tends to get stuck in local optima. Previous approaches using local search methods for protein folding simulation include Tabu Search [5,6], Simulated Annealing [7] and Population-based Local Search Method [8]. The main advantage of using local search methods is that they can quickly converge to better quality solutions, if not optimal, when efficient neighbourhood functions are employed.

Nevertheless, the uncertainty of stochastic search algorithms leads researchers to model and solve the problem using constraint programming. Yue and Dill [9] first presented a solution algorithm, called the *Constraint-based Hydrophobic Core Construction *(CHCC) method having faster runtime and claimed to give optimal solution. Later, Backofen & Will [10,11] made use of constraints over finite domains in both rectangular and *Face-Centered Cubic *(FCC) lattice using HP energy function. A *Constraint-based Protein Structure Prediction *(CPSP) tool was introduced, based on their method, which can predict the optimal structure of proteins having length up to 160 on FCC lattice in very short time [12]. Nevertheless, their approach is computationally intractable for more elaborate energy functions such as a 20 amino acid pairwise interaction energy function [13,14]. A *Two-Stage Optimization *approach was proposed later in [15] which uses CPSP tool to provide initial structure for local search procedure on FCC lattice and elaborate energy function. The two-stage optimization approach was reported to outperform simulated annealing-based local search procedure alone.

A better application of CP technique to protein folding is to encode the problem directly under constraint programming model and to search for the minimum energy structure. Palu et al. [16] showed heuristic approaches to protein folding problem using *Constraint Logic Programming *over finite domains (CLP(FD)). Despite satisfactory performances on small/medium sized instances, it was proved ineffective in scaling to larger instances of the problem [17]. Later, they developed a solver that can model the protein folding as a *Constraint Satisfaction Problem *(CSP) on 3D lattices and produced acceptable quality solutions for larger proteins [18]. In general, CP techniques guarantee to provide optimal solutions if the problem is modeled correctly. But as the solution space grows exponentially according to the problem size, the exponentially-increasing execution time is always a huge concern.

In this paper we combine local search and constraint programming approaches aiming the expected outcome of better quality solutions in acceptable execution time. We introduce a protein folding simulation procedure on FCC lattice that employs a CSP solver to generate neighbourhood states for a simulated annealing-based local search method. We use 20 amino acid pairwise interaction energy function introduced in [14]. The choice of the FCC lattice is motivated by the fact that it was shown to yield very good approximations of real protein structures [19]. Also it does not suffer from the bipartiteness of the cubic lattice, which allows interactions only between amino acids of opposite parity in the sequence. The hybrid approach introduced in this paper, produces considerably better solutions than pure constraint programming approach within practically feasible time-limit. It also produces better solutions within comparable time than those produced by local search employing well-established neighbourhood function.

## Results and discussion

This section reports the results obtained from running a collection of experiments using hybrid approach, pure constraint programming approach, pure local search approach and two-stage optimization approach (see **Background **Section). Preliminary tests are performed on 3 smaller proteins (namely 4RXN, 1ENH, 4PTI) to tune("learn") the various paramaeters. These parameters are then used to carry out the experiments on a larger set of proteins having length ranging from 54 to 74 including the learning set(the same used in [15]). The first column of each table contains official id of the protein assigned in the *protein data bank *(PDB) [20]. Since we completely omit the secondary structure information of the protein when solving the problem, these results are not comparable to those using secondary structure information (eg. [16-18]). All of the experiments have been performed on an Intel(R) Xeon(R) CPU E5440 at 2.83 GHz with 32 GB memory. The operating system is Linux CentOS and the compiler is gcc v.4.1.2 with the expensive optimization flags enabled. Since the selection of neighbourhood space is randomly guided in hybrid approach, different runs may lead to different solutions. So we executed 10 runs with the hybrid approach for each protein and report the results obtained on the best run. The number of iterations is set to 2000 for each LSA run in hybrid approach.

### Hybrid approach and pure CP approach

For each protein we executed 2 runs with the pure CP approach, one with complete enumeration search and another with *bounded block fail *(BBF) heuristic search. In Table 1, we report the search time and the final (best) energy value reached for each protein and compare them with the results from hybrid approach. The second column (N) denotes the number of amino acids in the protein. The 'Enumeration' column presents the best energy found using a complete exploration of search tree within the time limit. The 'BBF' column presents the same information using the BBF heuristic. Note that, for BBF heuristic the block size is set to 5 and the number of allowed failures for each block is set to 20. These parameters are explained in [18]. Finally the 'Hybrid' column shows the best energy found and the runtime after completing 2000 iterations using hybrid approach.

**Table 1:** Results: Comparison With Pure CP

		Enumeration		BBF heuristic		Hybrid	
Id	N	Energy	Time(limit)	Energy	Time(limit)	Energy	Time
4RXN	54	-14.52	5 h	-41.21	5 h	-168.076	1 h 19 m
1ENH	54	-24.058	5 h	-41.854	5 h	-157.062	1 h 16 m
4PTI	58	-22.811	5 h	-52.775	5 h	-213.778	1 h 30 m
2IGD	61	-19.598	5 h	-47.589	5 h	-186.696	1 h 13 m
1YPA	64	-22.831	5 h	-61.464	5 h	-258.709	1 h 08 m
1R69	69	-20.716	5 h	-57.491	5 h	-222.317	43 m
1CTF	74	-21.503	5 h	-30.697	5 h	-233.764	1 h 56 m

The energy values found with the hybrid approach are significantly better than the ones found with the pure CP approach in much lesser time. It was observed from our experiment that the time required for pure CP search to find the next best solutions from the current best ones are quite large, because of the fact that the search space is too big to explore and the search diverges. Even with the BBF heuristic, the performance is discouraging. On the other hand, we observed regular improvements in energy values during the SA run in hybrid approach.

### Hybrid approach and local search

For each protein sequence we performed 10 independent simulated annealing-based local search runs starting with random initial structures. Then we performed 10 independent runs for the two-stage optimization approach outlined in [15]. The number of iterations for the local search stages were set to 1,500,000. We used *pull move sets *[6] as neighbourhood functions for the local search, which were shown to be very efficient for protein folding simulation [5,6]. In Table 2, we compare search time and best energy value results from various approaches. The 'LSA' column presents the best energy value found using pure logarithmic simulated annealing procedure. The 'Two-stage' and 'Hybrid' columns present the best energy value and the time to reach this state using two-stage optimization approach and hybrid approach respectively. Note that, 'Hybrid-Time'column in Table 2 reports the minimum runtime to reach the best energy value, whereas 'Hybrid-Time'column in Table 1 reports the total runtime to complete 2000 iterations.

**Table 2:** Results: Comparison With Local Search.

	LSA	Two-stage			Hybrid
Id	Energy	Energy	Time	Energy	Time
4RXN	-165.401	-167.781	10 m 51 s	-168.076	1 h 05 m
1ENH	-152.747	-153.098	2 m 33 s	-157.062	1 h 02 m
4PTI	-215.698	-212.500	6 m 21 s	-213.778	1 h 20 m
2IGD	-180.893	-183.205	2 m 37 s	-186.696	55 m
1YPA	-256.017	-257.81	16 m 54 s	-258.709	42 m
1R69	-215.166	-219.402	14 m 42 s	-222.317	35 m
1CTF	-228.921	-233.86	11 m 12 s	-233.764	1 h 36 m

One can observe that the energy values found with the two-stage optimization are generally better than pure local search whereas hybrid approach gives better energy values than two-stage optimization. It was reported in [15] that two-stage optimization generally reaches better solutions in fewer number of iterations than local search alone. Therefore we compared the runtime of hybrid approach with two-stage optimization only. We can see that, hybrid approach produces better final energy values than two-stage optimization but takes longer time to do so. The reason is explained in **Methods **Section. Still the runtime of hybrid approach is within acceptable range given the quality of the solution it generates.

## Conclusion

The purpose of our present work was to demonstrate that protein folding problem on FCC lattice with elaborate energy functions can be approached by hybrid use of constraint programming and local search; and by making the best use of both techniques, hybrid approach can outperform the only use of constraint programming and local search, in terms of quality of solutions and execution time. We first modeled a basic protein folding problem on FCC lattice with 20 amino acids pairwise interaction energy function into the constraint programming framework of COLA solver. Then we integrated this CP model with a simulated annealing-based local search in such a way that the COLA solver can provide random neighbourhood states. We kept the modeling as simple as possible by using an easy definition of contact distance and allowing all possible angles on FCC lattice. Our tests confirmed that the hybridization of these techniques generates siginificantly better solution with better execution time than the pure constraint programming model. Our tests also showed that hybrid approach results in better solution compared to simulated annealing with pull move set but it takes longer execution time.

We need to work on refining the parameters reported in Table 3 that control the search space and execution time. It was found out during the preliminary tests that only use of the shorter subchain length (eg. *l *= 6) quickly leads to solutions slightly worse than those reported in Table 2. But the process then got stuck into local minima and longer *l *had to be chosen in order to escape from there increasing the runtime considerably. We can look for better escape strategies that will not contribute significantly to the execution time.

**Table 3:** Parameter Settings. Combination of parameters used during CSP solving part in each iteration of local search. *l *denotes the length of subchain selected for perturbation, *b *denotes the domain dilation parameter and (*l*) denotes the probability of selecting length *l*.

*l*	*b*	**(*l*)**
7	3	0.5
9	2	0.3
11	1	0.15
13	1	0.5

Nevertheless, based on the encouraging results we obtained from simple modeling, we can conclude that hybrid approach would be a good idea to attack the protein folding problem for more realistic models. Tertiary structures often contain local 3D rigid conformations known as *secondary structure *(eg. *α*-helices, *β*-sheets). We can use these secondary structure information in order to predict more realistic conformations since the contact based energy function is not sufficient enough to reproduce local arrangements such as helices and/or sheets. Using secondary structure information, obtained through neural network prediction, not only helps to predict realistic tertiary structures but also improves the execution time significantly by over-constraining the problem. We can also include additional constraints derived from known chemical and physical properties [21] to speed-up execution. As future work, we plan to incorporate these constraints to our hybrid approach aiming to get four distinct targets. Firstly, we will get more realistic final conformations. Secondly, we will get it in lesser execution time. Thirdly, we will predict larger instances of proteins. Finally, we can compare the effectiveness of our approach over existing COLA implemetation of [18]

Last but not least, the idea of modeling a problem in constraint programming framework and then using it in a local search algorithm as neighbourhood function can be an interesting approach to solve other hard combinatorial optimization problems. Almost all optimization problems in biology are NP-hard and many real problems have beautiful natural combinatorial formulations. The application of hybrid approach can be useful in this particular context.

## Methods

### The protein folding problem

The *primary structure *of a protein is comprised of a sequence of *n *amino acids, i.e., *s *= {*s*_1_, ..., *s*_*n*_} where each *s*_*i *_∈ .  is the alphabet of amino acids and || = 20. Given a lattice model ℒ, the 3D conformation of protein is the assignment of amino acids in the lattice points such that adjacent positions of *s *remain adjacent in the lattice and no two amino acids overlap. The protein tends to reach a 3D conformation with minimum free energy, which is called its *tertiary structure*. One idea of determining the free energy associated with a particular conformation is to assign energy to pairs of amino acids on the lattice within a predefined distance (formalized as *contacts*) and then summing all up. The objective of the protein folding problem is to determine the 3D conformation from primary structure such that the free energy is minimized.

#### Lattice and energy model

The domain of FCC lattice consists of points (*x*, *y*, *z*) ∈ ℤ such that *x *+ *y *+ *z *is even. Two FCC points *P*_*i*_(*x*_*i*_, *y*_*i*_, *z*_*i*_) and *P*_*j*_(*x*_*j*_, *y*_*j*_, *z*_*j*_) are *adjacent *if and only if |*x*_*i *_- *x*_*j*_| ≤ 1, |*y*_*i *_- *y*_*j*_| ≤ 1, |*z*_*i *_- *z*_*j*_| ≤ 1 and |*x*_*i *_- *x*_*j*_| + |*y*_*i *_- *y*_*j*_| + |*z*_*i *_- *z*_*j*_| = 2. Each FCC lattice point is adjacent to 12 neighbouring points and three consecutive adjacent points forms one of these four angles 60°, 90°, 120°, 180°. Observe that, for lattice units, it holds that |*x*_*i *_- *x*_*j*_| + |*y*_*i *_- *y*_*j*_| + |*z*_*i *_- *z*_*j*_| = 2. Two amino acids *s*_*i *_and *s*_*j *_in lattice positions *P*_*i *_and *P*_*j*_, respectively, are said to be in *contact*, i.e., *contact*(*P*_*i*_, *P*_*j*_), if and only if they are not adjacent in the primary sequence, i.e., |*i *- *j*| > 1 and |*x*_*i *_- *x*_*j*_| + |*y*_*i *_- *y*_*j*_| + |*z*_*i *_- *z*_*j*_| = 2.

In [14], the authors developed table of empirical contact potential that points out the energy contributions associated to pairs of amino acids *s*_*i *_and *s*_*j *_in contact, described by the commutative function *energy*(*s*_*i*_, *s*_*j*_). These contributions are developed from statistical methods applied to structures obtained from X-rays and NMR experiments.

#### Mathematical formalization

Given the amino acid sequence *s*, a *conformation ϕ *of *s *in FCC lattice is an injective function *ϕ*: {1... *n*} → ℒ such that *ϕ *(*s*_*i*_) and *φ*(*s*_*i*+1_) are *adjacent *for *i *= 1... *n *- 1 and *ϕ*(*s*_*i*_) ≠ *ϕ*(*s*_*j*_) for *i *≠ *j *to avoid overlapping.

The *protein folding problem *can then be formalized as an optimization problem for finding the conformation *ϕ *of *s *such that the following energy function is minimized:(1)

### CP framework

Modeling the protein folding problem on FCC lattice leads to the following structural constraints.

• **adjacent property**: Adjacent amino acids in the primary sequence are mapped to adjacent lattice points. For each *i *∈ {1... *n *- 1}, |*x*_*i *_- *x*_*i*+1_| + |*y*_*i *_- *y*_*i*+1_| + |*z*_*i *_- *z*_*i*+1_| = 2

• **non-overlap property**: Two non-adjacent amino acids in the primary sequence must not occupy the same lattice point and must be separated by at least one lattice unit. For each *i*, *j *∈ {1... *n*} and |*i *- *j*| > 1, |*x*_*i *_- *x*_*j*_| + |*y*_*i *_-*y*_*j*_| + |*z*_*i *_- *z*_*j*_| ≥ 2

The protein folding problem is effectively a *Constraint Satisfaction Problem *(CSP) defined by the constraints above with an objective energy function (Equation 1) to be minimized. Authors in [16,18] added some extra constraints to disallow angles of 60° and 180° for three consecutive amino acids. Also their definition of contact distance is somewhat different from us. This study is, however, intended to measure the effectiveness of hybrid approach over local search and pure constraint programming approaches. Protein folding simulation results have been reported in the literature with local search using efficient neighbourhood function that allows all the possible angles for three consecutive amino acids in FCC lattice [6,15]. These definition of angles and contact distance are used in our work in order to make valid comparisons.

#### COLA solver

COLA is an ad-hoc constraint solver on discrete 3-dimentional crystal lattices developed by Palu et al. [18]. The solver allows user to define lattice variables with associated domains, constraints over them and to search the space of admissible solutions.

Given a sequence *s *and lattice ℒ, the *lattice variable V*_*i *_represents the lattice point (*x*_*i*_, *y*_*i*_, *z*_*i*_) of amino acid *s*_*i*_. A *domain D *is described by a pair of points (*D*, ), where *D*= (*D*_*x*_, *D*_*y*_, *D*_*z*_) ∈ ℤ and . *D *implicitly defines a *box*:

Each variable *V *is associated to a *domain D*^*V*^described by a pair of points (*D*^*V*^, ). *V *is *admissible *if *Box*(*D*^*V*^) ≠ Φ. *V *is *ground *if it is admissible and *D*^*V *^= .

A set of primitive binary constraints is defined in the form of *C*(*V*_*i*_, *V*_*j*_, *d*) over two variables *V*_*i *_and *V*_*j*_, based on spatial distances *d *∈ ℕ. A *CSP *on the variables *V*_1_, ..., *V*_*n *_with domains  is a set of constraints of above-mentioned forms. A solution of the CSP is an assignment of lattice points to the variables *V*_1_, ..., *V*_*n *_within corresponding domains and satisfying all the binary constraints.

The solver is modeled in a way that separates constrain phase from search phase, thereby not allowing addition of new variables or constraints during search phase. The solver uses a combination of consistency techniques and systematic search to guide the solving process. Standard *backtracking+propagation *search procedure [22] is implemented to explore the search tree efficiently. The detailed description on COLA solver can be found in [18].

### LS framework

*Simulated annealing *(SA) was introduced as an optimization tool independently in [23,24]; see also [25]. The underlying algorithm acts within a solution space in accordance with a specific neighbourhood structure, where the transition steps are controlled by the objective function. The solution space for the protein folding problem consists of all the possible *self-avoiding walks *(SAW) on the FCC lattice. The objective function to be minimized here is given by the Equation 1. A logarithmic cooling schedule is employed which was shown in [26] to converge to optimal solutions. In the context of local search methods, it is important to employ an efficient neighbourhood function that determines the overall performance in terms of run-time and final energy value. We use CP technique described in **CP framework **Section to generate the neighbourhood for LS.

### CP as neighbourhood generator for LS

A CSP can be built for a given protein sequence that enumerates all conformations satisfying structural constraints of protein in a given lattice. The procedure is basically an exploration of exponentially large search tree. With the most efficient propagation+backtracking technique, such an enumeration requires huge amount of time even for the medium sized instance. The size of the solution space for the protein folding problem is the number of self-avoiding walks on the FCC lattice that can be approximated by the formula (see [27]).(2)

Without the presence of additional constraints (eg. secondary structure information, not allowing certain angles in the folding etc. [16,18]), exploring this exponential search tree for the best energy value is infeasible.

Instead of exploring the complete search tree, we propose to explore a small part of it at a time and use this information for the next step. In each iteration of a standard local search procedure, the CSP solver is asked to enumerate all the possible neighbours of the current conformations by keeping certain parts (variables) of it fixed to its current lattice positions and allowing a small part to change their positions. The best neighbouring conformation found this way will be used for the next iteration.

We denote small parts of the protein sequence as *subchains *for the rest of the discussion. The *Logarithmic simulated annealing *(LSA) procedure starts with an initial conformation, i.e., all lattice variables [*V*_1_, ..., *V*_*n*_] are ground. Then in each iteration of LSA, a CSP solver generates random neighbourhood in the following way:

**Step 1**. Randomly select a lattice variable *V*_*i*_.

**Step 2**. Randomly select the length of the subchain *l *∈ {7, 9, 11, 13} to be perturbed.

**Step 3**. Keep the two parts of the conformation unchanged, namely lattice variable sets [*V*_1_, ... *V*_*i*_] and *V*_*i*+*l*+1_... *V*_*n*_. These variables remain fixed (ground) to their current values.

**Step 4**. For each variable *V*_*j *_with lattice point (*x*_*j*_, *y*_*j*_, *z*_*j*_) in the subchain [*V*_*i*+1_, ... *v*_*i*+*l*_], we redefine the domains  as follows:  and .

Here 1 ≤ *b *≤ 3 is the *domain dilation *parameter which controls the size of the domain.

**Step 5**. Let CSP solver enumerate all the conformations based on current variable and domain definitions. Select the conformation with minimum energy.

In short, given a protein sequence and its current conformation having certain parts fixed, we ask CSP to find all the possible conformations by altering the remaining part of the conformation and also not changing the current conformation too much. The convergence of the solution towards lower energy state depends on the efficiency of the local search procedure (i.e., cooling schedule) employed and careful choice of subchain length (*l*) and domain dilation (*b*) parameters.

The choice of initial conformation is particularly important for our approach. It is not only recommended, but also absolutely necessary to start with a somewhat random "compact" structure irrespective of the energy value. At the initial stage of the search, the compact structure allows CSP solver to generate "good" neighbourhood conformations that help local search algorithm to converge quickly to conformations with lower energy. Otherwise, if we start with a "flat" initial conformation like a straight sheet-like structure, it take ages for local search to get to even moderately compact structure. Since the purpose of CSP solver is to rearrange a small subchain within a bounded box (defined by the domains of the corresponding variables), the shape of the box directly effects the possible number of rearrangements, i.e., the number of self avoiding walks within the box with two fixed end-points. It is observed that, for a curve-like subchain structure, the number of SAW's are higher, whereas for a sheet-like subchain structure, the number of SAW's are lower. Therefore, in our implementation of local search, the initialization procedure generates a compact initial conformation by iterating the following steps as long as amino acids are available to be placed.

**Step 1**. Apply consecutive forward-left and forward-right move for 5 times.

**Step 2**. Go up by applying consecutive left-up and right-up move once.

**Step 3**. Apply consecutive backward-right and backward-left move for 5 times.

**Step 4**. Go up by applying consecutive left-up and right-up move once again.

**Figure 1:**
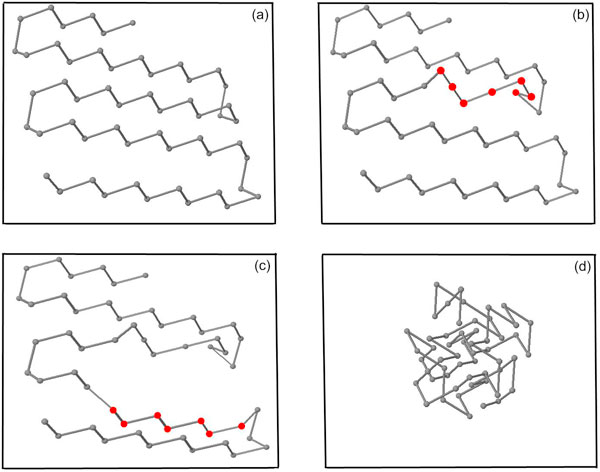
**Conformations for 1ENH**. (a) Initial conformation with energy value -0.885 (b) Conformation with energy value -19.154 after first iteration (c) Conformation with energy value -29.006 after second iteration (d) Final conformation with energy value -157.062 after 2000 iterations. The circles represent the amino acids. The red circles in (b) and (c) represent the subchain perturbed during the local search iteration.

Figure 1(a) illustrates such an initial conformation for a protein having length of 54. Figure 1(b-c) shows the conformations obtained from two consecutive hybrid local search iterations using this initial conformation. Figure 1(d) depicts the final conformation.

### Modification to COLA

COLA is a public-domain solver available for download in [28]. It contains the implementation of protein folding problem on FCC lattice with additional constraints and different notions of contact distance as mentioned earlier. For the sake of our study, we remove those additional constraints and modify the contact definition as well. The major part of modification, though, has to be done in the search procedure. In the original implementation, the search procedure starts with the leftmost variable with all the variables non-ground and not labeled. The search continues by assigning values to variables (*labeling*) one after another according to one of the two *variable selection *strategies (*leftmost *or *first-fail*) employing standard combination of consistency checking and propagation techniques. Once all the variables are labeled after *n *level of branching, the search backtracks to the last modified variables and assign new values to it (if available). This way the whole search space is explored systematically. In our case, most of the variables (*n *-*l*) are set ground at the beginning of search and the *l *non-ground variables are assigned new domains. Therefore we modify the search procedure to make it start from the leftmost non-ground variable and continue the process till the rightmost non-ground variable using leftmost variable selection strategy that explores only *l *level of branching. While *bounded block fails *(BBF) heuristic for solution searching over the whole solution space is more efficient [18], a complete enumeration is found to be more appropriate for our purpose, since we have to deal with only a small block of variables during each search phase. For the same reason, the choice of variable selection strategy is immaterial too. Therefore, we implement *leftmost *variable selection strategy with complete enumeration of search tree for our approach.

This modified search procedure is integrated into LSA to work as neighbourhood generator as described in **CP as neighbourhood generator for LS **Section. The choice of the range for subchain length, *l *is purely empirical. It is observed that CSP performs well with the smaller choice of *l *and tends to give better solution in shorter time than with longer *l*. On the other hand, though longer *l *results in increasing search time, they are vital for allowing local search to escape from local minima at times. Therefore, when selecting the length of the subchain to be altered, randomness is skewed in such a manner that shorter *l *are selected more than longer *l*. Since the choice of *l *and *b *directly effects the size of the search space (in effect runtime), we empirically find the acceptable combination of *l*, *b *and distribution of *l *((*l*)) that significantly speed-up the CSP solving phase (see Table 3).

The precise mathematical anaylsis for calculating the size of the search space explored by hybrid approach is complex and yet to be explained by us. It could be explained best by finding the number of self avoiding walks within a bounded box on FCC lattice when two end-points are fixed, which is a complex problem itself and deserves attention from mathematicians. In general, the upper bound of the search space in each iteration of the hybrid local search algorithm is close to a number 10^*l*^. *l *is the length of the subchain to be perturbed and the number of effective neighbouring positions for a given variable are approximated by 10. The tuning of parameters *l *and (*l*) allows us to restrict the search space from a number close to 10^*n *^(see Equation 2) to a number close to *I*_h_* 10^8.5 ^where *I*_*h *_is the number of iterations used for hybrid LSA procedure. The introduction of domain dilation parameter *b *further reduces the search space by reducing the effective neighbouring positions to a number less than 10. It explains why hybrid approach gains significant speed-up over pure CP approach. The runtime difference between pure simulated annealing and hybrid simulated annealing can be explained similarly by the fact that pure simulated annealing explores a smaller search space of size *I*_*ls*_, where *I*_*ls *_is the number of maximum iterations used in the algorithm.

## Competing interests

The authors declare that they have no competing interests.

## Authors' contributions

KS conceived the original idea of hybridization. ADU developed the systematic model. Implementation and experimentation were carried out by ADU. KS initiated, supervised and coordinated the work. Both authors contributed significantly in writing the manuscript and approved the final version.
